# Three-year results from a randomized trial of lumbar discectomy with annulus fibrosus occlusion in patients at high risk for reherniation

**DOI:** 10.1007/s00701-019-03948-8

**Published:** 2019-05-15

**Authors:** Jenny C. Kienzler, Peter Douglas Klassen, Larry E. Miller, Richard Assaker, Volkmar Heidecke, Susanne Fröhlich, Claudius Thomé, On behalf of the Annular Closure RCT Study Group

**Affiliations:** 1 Department of Neurosurgery, Kantonsspital Aarau, Tellstrasse 1, 5001 Aarau, Switzerland; 2 Department of Neurosurgery, St. Bonifatius Hospital, Lingen, Germany; 3 Miller Scientific Consulting, Inc., Asheville, NC USA; 4 Department of Neurosurgery, Centre Hospitalier Régional Universitaire de Lille, Lille, France; 5 Department of Neurosurgery, Klinikum Augsburg, Augsburg, Germany; 6 Department of Orthopedics, Universität Rostock, Rostock, Germany; 7 Department of Neurosurgery, Medical University Innsbruck, Innsbruck, Austria

**Keywords:** Annular closure device, Annulus fibrosus, Disc herniation, Lumbar discectomy, Randomized controlled trial, Sciatica

## Abstract

**Background:**

A larger defect in the annulus fibrosus following lumbar discectomy is a well-known risk factor for reherniation. Procedures intended to prevent reherniation by sealing or occluding the annular defect warrant study in high-risk patients. This study sought to determine 3-year results of lumbar discectomy with a bone-anchored annular closure device (ACD) or lumbar discectomy only (controls) in patients at high risk for reherniation.

**Methods:**

This multicenter randomized trial enrolled patients with sciatica due to lumbar intervertebral disc herniation who failed conservative treatment. Patients with large annular defects after lumbar limited microdiscectomy were intraoperatively randomly assigned to receive ACD or control. Clinical and imaging follow-up was performed at routine intervals over 3 years. Main outcomes included rate of reherniations, reoperations, and endplate changes; leg and back pain scores on a visual analogue scale; Oswestry Disability Index (ODI); Physical Component Summary (PCS) and Mental Component Summary (MCS) scores from the SF-36; and adverse events adjudicated by a data safety monitoring board.

**Results:**

Among 554 randomized patients, the modified intent-to-treat population consisted of 272 patients in which ACD implantation was attempted and 278 receiving control; device implantation was not attempted in 4 patients assigned to ACD. Outcomes at 3 years favored ACD for symptomatic reherniation (14.8% vs. 29.5%; *P* < 0.001), reoperation (11.0% vs. 19.3%; *P* = 0.007), leg pain (21 vs. 30; *P* < 0.01), back pain (23 vs. 30; *P* = 0.01), ODI (18 vs. 23; *P* = 0.02), PCS (47 vs. 44; *P* < 0.01), and MCS (52 vs. 49; *P* < 0.01). The frequency of all-cause serious adverse events was comparable between groups (42.3% vs. 44.5%; *P* = 0.61).

**Conclusions:**

The addition of a bone-anchored ACD in patients with large annular defects following lumbar discectomy reduces the risk of reherniation and reoperation, and has a similar safety profile over 3-year follow-up compared with lumbar limited discectomy only.

**Trial registration:**

ClinicalTrials.gov NCT01283438

**Electronic supplementary material:**

The online version of this article (10.1007/s00701-019-03948-8) contains supplementary material, which is available to authorized users.

## Introduction

Lumbar discectomy is an effective surgery for chronic sciatica secondary to intervertebral disc herniation. However, symptom recurrence following surgery is a common risk that is influenced by patient- and surgery-related factors such as male sex, disc degeneration, and large post-surgical annular defect size [8]. Patients with a post-surgical annular defect of at least 6 mm in width have a reherniation risk nearly three times higher compared with patients with smaller defects [13]. Consequently, adjunctive treatments intended to contain the nucleus pulposus within the disc space following discectomy have been studied. So far, results have been disappointing with sutures, fibrin glue, and polyethylene plug due to the failure of these materials to chronically withstand high intradiscal pressures [1, 2, 5, 7]. An implantable device intended to provide a more durable repair has been developed. This annular closure device (ACD) anchors into the adjacent vertebral body and occludes the damaged annulus fibrosus with a polymer mesh. Results from case series [4, 11, 12, 15] and a recent randomized trial [17] with this device have demonstrated clinically important reductions in reherniation and reoperation rates through 2 years. In this report, we extend these findings by presenting 3-year results from a randomized trial of 554 patients who received lumbar discectomy with bone-anchored annular closure or lumbar microdiscectomy only.

## Materials and methods

This was a multicenter, multinational, randomized controlled trial to determine the effectiveness and safety of lumbar discectomy with a bone-anchored implant designed to provide annulus fibrosus occlusion in patients at high risk for reherniation (ClinicalTrials.gov NCT01283438). Local ethics committees reviewed and approved the protocol. All participants provided written informed consent before trial participation. The design [9] and 2-year primary endpoint results [17] of this trial have previously been described. This report presents 3-year clinical and radiographic results from the trial.

Preoperative imaging included magnetic resonance imaging (MRI) with T1- and T2-weighted axial and sagittal images, index-level low-dose multiplanar computed tomography (CT), and anteroposterior/lateral and flexion/extension x-rays. Important eligibility criteria for the study included diagnosis of a single-level lumbar disc herniation identified on preoperative imaging, and concurrent clinical findings (positive straight leg raise or femoral stretch test) with leg pain (≥ 40 on a 0–100 visual analogue scale) that were not responsive to at least 6 weeks of conservative treatment, and at least moderate disability (≥ 40 on the Oswestry Disability Index). A complete list of study entry criteria is provided in Supplement Table 1. Patients meeting these criteria were treated with limited lumbar microdiscectomy but were not yet enrolled in the study. When the discectomy procedure was completed, the final study entry criterion was applied. Patients with a large defect in the annulus fibrosus, defined as 4–6-mm height and 6–10-mm width, were enrolled in the study and randomly allocated (1:1) to receive discectomy only (controls) or to additionally receive a bone-anchored ACD with a mesh occlusion component (Barricaid, Intrinsic Therapeutics, Woburn, MA, USA) designed to physically block the annular defect. Patients with large annular defects were specifically targeted for this trial given their well-known high risk of reherniation after lumbar discectomy [13]. Following randomization, no additional disc material was removed in either treatment group. When annular defects of ineligible size were intraoperatively identified, the discectomy procedure was completed in the usual fashion and patients were discontinued from the study.

Clinical and imaging follow-up occurred at 6 weeks, 3 months, 6 months, and at annual intervals for 3 years and included MRI, low-dose CT, and AP/lateral and flexion/extension x-rays. Patients in this study will remain in follow-up for 5 years. A schematic that lists the clinical and imaging tests performed at each study interval is provided in Supplement Table 2**.** Symptomatic reherniation was defined as a reherniation (protrusion, extrusion, or sequestration) that was confirmed during a reoperation, or identified on imaging with associated recurrent or new lumbar pain, leg pain, or neurological deficit. Reoperation included any repeat procedure at the index level of herniation including discectomy, supplemental fixation, fusion, or device explant. Key radiographic assessment by x-ray and CT included disc height, device status, and vertebral endplate changes (VEPC). Imaging evaluations were read by an independent core laboratory radiologist who was blinded to clinical outcomes. Clinical outcome parameters included leg and back pain severity, Oswestry Disability Index (ODI), SF-36 Physical Component Summary (PCS) score, and SF-36 Mental Component Summary (MCS) score. The minimal important differences (MID) were defined as a ≥ 20-point decrease from baseline for leg pain [14], ≥ 20-point decrease from baseline for back pain [14], ≥ 15-point decrease from baseline for ODI [6], ≥ 5.7-point increase from baseline for PCS [18], and ≥ 6.3-point increase from baseline for MCS [18]. Neurological status and adverse events were assessed at each follow-up visit. Investigators classified adverse events by seriousness and relation to the device or procedure. Neither patients, surgeons, outcome assessors nor imaging core laboratory readers were blinded to group allocation, with the exception of patients in the Netherlands due to regional regulations. An independent data safety monitoring board provided safety oversight during the study and adjudicated all adverse events.

Statistical analyses were performed on a modified intention-to-treat population consisting of randomized patients in whom the intended procedure was attempted. Preoperative group characteristics were reported as mean and standard deviation for continuous variables, and count and percentage for categorical variables. Group comparisons were performed with Student’s *t* test for continuous data, Fisher’s exact test for categorical data, and log-rank tests for survival data. In patients who underwent a reoperation prior to the 3-year follow visit, leg pain, back pain, ODI, PCS, and MCS values at 3 years were substituted with baseline values. Statistical significance was set at *P* < 0.05 and hypothesis testing was two-sided. Statistical analyses were performed using SAS v9.4 (SAS Institute, Cary, NC, USA) and R v3.3.2 (R Foundation for Statistical Computing, Vienna, Austria).

## Results

**Fig. 1:**
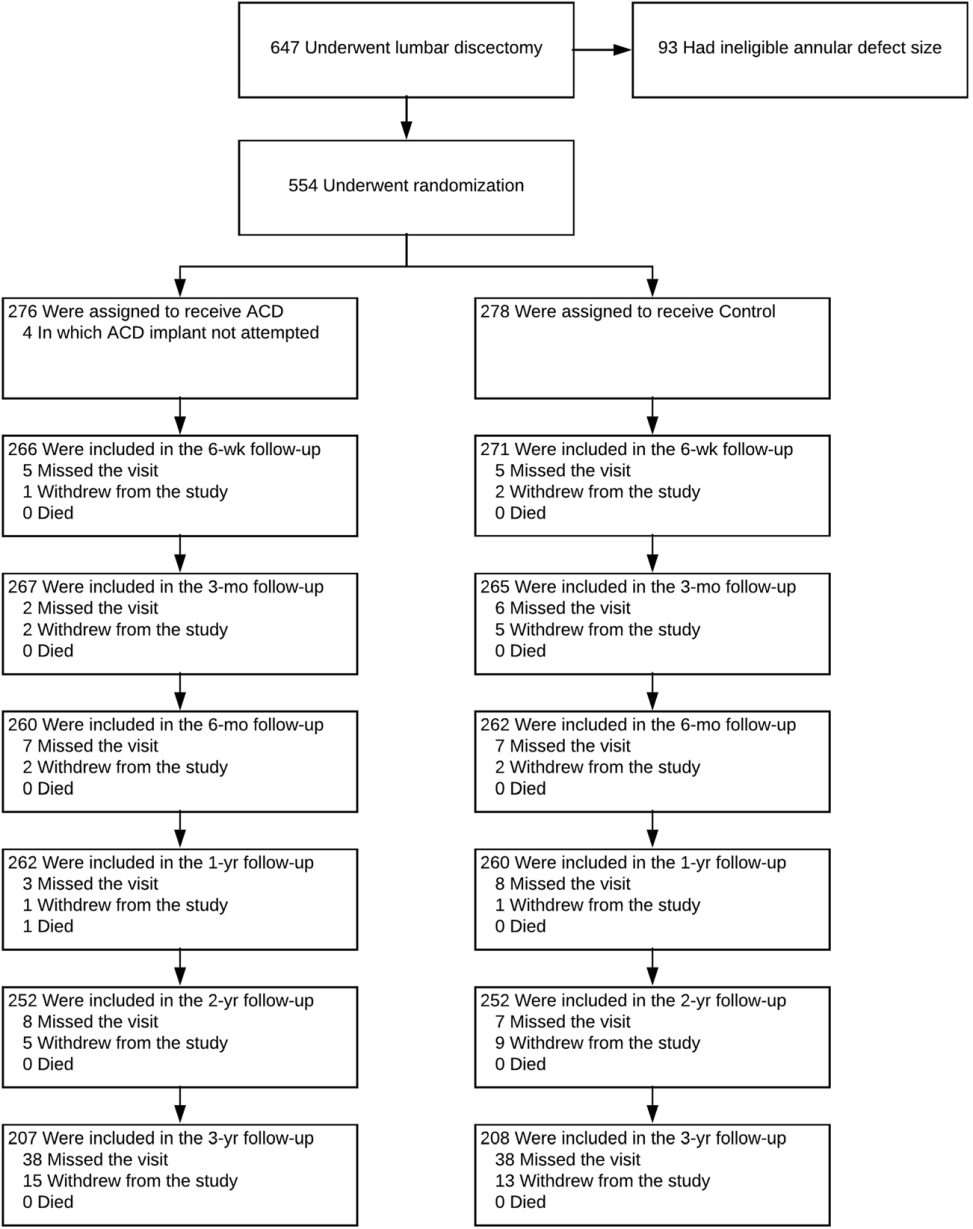
Enrollment and randomization of patients. Among 554 randomized patients, 276 were allocated to annular closure device (ACD) and 278 to control. Owing to 4 patients in whom ACD implant was not attempted, the modified intent-to-treat population consisted of 272 patients with attempted ACD implant and 278 patients assigned to control. Compliance with clinical follow-up at 3 years was 76% with ACD and 75% with controls

A total of 554 patients were randomly allocated to receive ACD (*n* = 276) or control (*n* = 278) at 21 hospitals (Study Group Appendix Table 4)) between December 2010 and October 2014. Annular closure device implantation was not attempted in four patients because of the close proximity of the nerve root and the associated potential risk. Therefore, the modified intention-to-treat population included 272 ACD patients and 278 control patients. Among the ACD group were 5 patients in which the device was unsuccessfully implanted due to incomplete entry of the occlusion mesh into the disc space (*n* = 4) or nerve root injury (*n* = 1). The mean age of all enrolled patients was 43 ± 11 years and 59% were men. Disc herniation was most commonly identified at L5-S1 (56%) or L4-L5 (41%). Patients typically presented with severe leg pain (overall mean 81 ± 15), severe disability (overall mean ODI 59 ± 13), and moderate back pain (overall mean 56 ± 31) (Table 1). Overall, 415 (75%) patients (207 ACD, 208 control) returned for clinical follow-up at 3 years (Fig. 1).

**Table 1:** Baseline patient characteristics

Characteristic	ACD (*n* = 272)	Control (*n* = 278)
Age (year)	43 ± 11	44 ± 10
Male sex	156 (57)	171 (62)
Body mass index (kg/m^2^)	26 ± 4	26 ± 4
Smoking history	173 (64)	175 (63)
Leg pain severity (mm)	81 ± 15	81 ± 15
Back pain severity (mm)	57 ± 30	56 ± 31
Oswestry Disability Index	59 ± 12	58 ± 14
Index level		
L2-L3	2 (1)	1 (< 1)
L3-L4	8 (3)	5 (2)
L4-L5	123 (45)	101 (36)
L5-S1	139 (51)	171 (62)
Spondylolisthesis, grade 1	6 (2)	8 (3)
Disc height (mm)	8.9 ± 2.1	8.9 ± 2.2
Extrusion or sequestration	201 (74)	201 (72)

Values are mean±standard deviation or count (percentage)

*ACD*, annular closure device

**Fig. 2:**
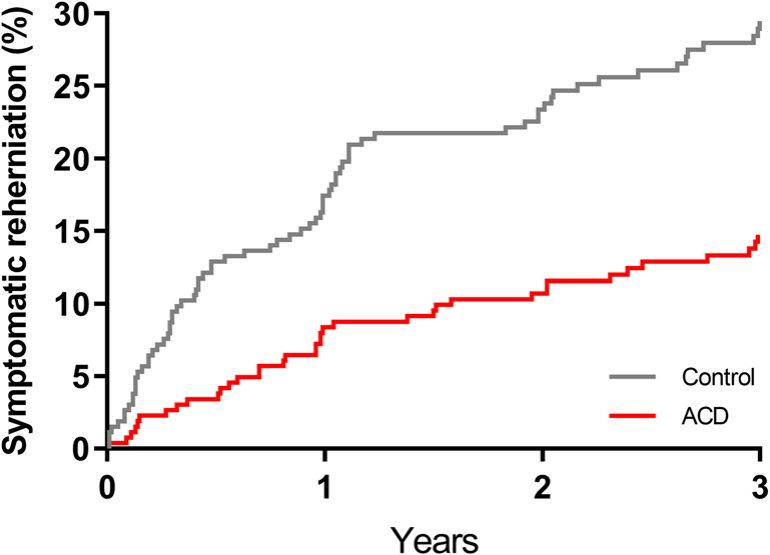
Cumulative rate of symptomatic index level reherniation through 3 years. Cumulative event rates were 14.8% for annular closure device (ACD) and 29.5% for control (log-rank *P* < 0.001)

**Fig. 3:**
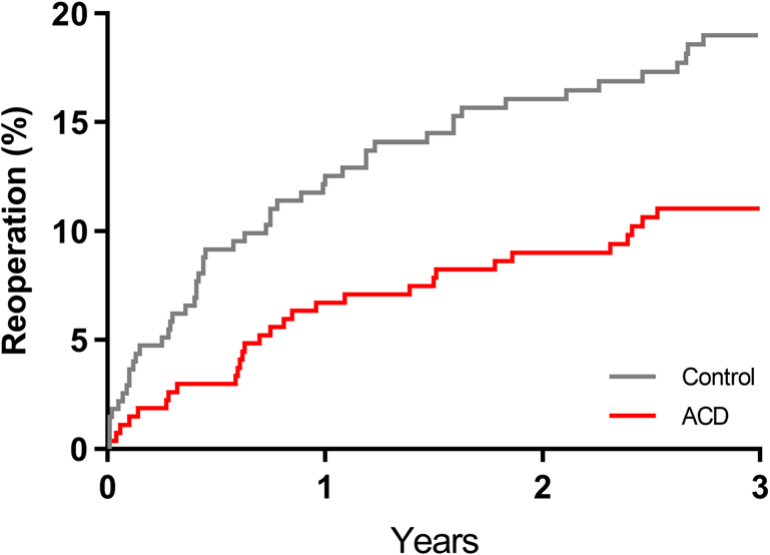
Cumulative rate of index level reoperation through 3 years. Cumulative event rates were 11.0% for annular closure device (ACD) and 19.3% for control (log-rank *P* < 0.001)

The risk of symptom recurrence through 3 years was lower in patients treated with ACD versus controls; the cumulative incidence of symptomatic reherniation was 8.4% vs. 17.4% at 1 year, 10.7% vs. 23.4% at 2 years, and 14.8% vs. 29.5% at 3 years (log-rank *P* < 0.001 at 3 years) (Fig. 2). Similarly, reoperations were less frequent in the ACD group, with cumulative reoperation rates of 6.7% vs. 12.9% at 1 year, 9.0% vs. 16.4% at 2 years, and 11.0% vs. 19.3% at 3 years (log-rank *P* < 0.001 at 3 years) (Fig. 3). There were 38 reoperations in 29 ACD patients and 70 reoperations in 51 control patients. Reoperation strategies between groups were similar with repeat discectomy performed most frequently (Table 2).

**Table 2:** Types of reoperations performed through 3 years

Characteristic	ACD	Control
Discectomy	14	41
Fusion	14	14
ACD removal only	5	NA
Decompression procedure	3	2
Wound complication	1	6
Dural tear repair	1	0
Discectomy with ACD implant	NA	4
Hematoma drainage	0	3
Total	38	70

*ACD*, annular closure device; *NA*, not applicable

**Fig. 4:**
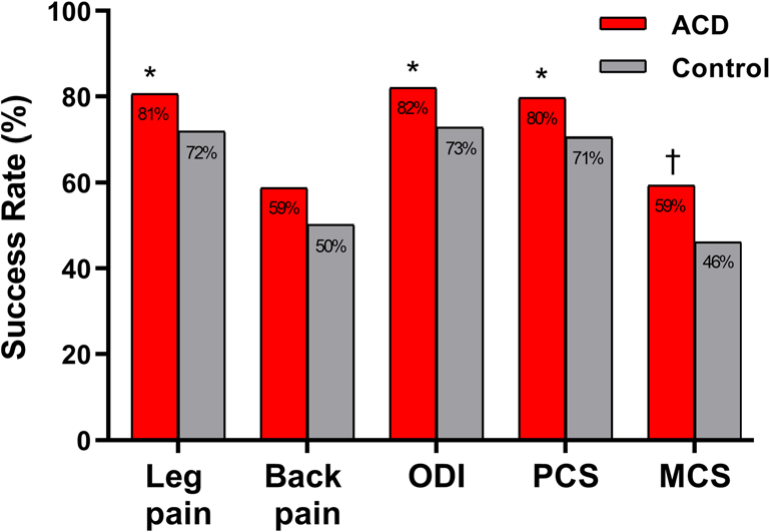
Percentage of patients achieving the minimal clinically important difference (MID) in patient-reported outcomes through 3 years. MID defined as improvement from baseline of at least 20 points for leg pain, 20 points for back pain, 15 points for Oswestry Disability Index (ODI), 5.7 points for Physical Component Score (PCS), and 6.3 points for Mental Component Score (MCS), respectively (all reported on 0–100 scale). Statistical significance between treatment groups denoted as *P < 0.05 or †*P* < 0.01. ACD, annular closure device

Patient-reported outcomes favored those treated with the ACD. Comparing ACD with controls, mean values at 3 years were 21 vs. 30 (mean difference = − 8, 95% CI = − 2 to − 15, *P* < 0.01) for leg pain, 23 vs. 30 (mean difference = − 7, 95% CI = − 1 to − 12, *P* = 0.01) for back pain, and 18 vs. 23 (mean difference = − 5, 95% CI = − 1 to − 9, *P* = 0.02) for ODI. Health-related quality of life scores at 3 years was higher in the ACD group; PCS scores were 47 vs. 44 (mean difference = 3, 95% CI = 1 to 5, *P* < 0.01) and MCS scores were 52 vs. 49 (mean difference = 3, 95% CI = 1 to 5, *P* < 0.01). The percentage of patients achieving the MID was statistically greater in the ACD group for leg pain, ODI, PCS, and MCS; no statistical difference between groups was noted for back pain (*P* = 0.08) (Fig. 4).

Disc height decreased by approximately 30% in each group relative to baseline. Average disc height at 3 years was 6.3 ± 2.2 mm with ACD and 6.4 ± 2.2 mm with controls (*P* = 0.64). Vertebral endplate changes were identified by the imaging core laboratory in 89% of ACD patients and in 41% of controls (*P* < 0.001). The median VEPC areas at 2- and 3-year follow-up were 0.42 cm^3^ and 0.49 cm^3^ with ACD, 0.29 cm^2^ and 0.42 cm^2^ for controls. Neither the frequency, area, nor growth trajectory of VEPC was associated with clinical sequelae.

Neurological function deterioration relative to baseline was reported in 2.0% of ACD patients and 4.3% of controls at 3 years (*P* = 0.26). Serious adverse events related to the device or procedure occurred in 10.7% of the ACD group and in 18.7% of controls (*P* = 0.008), and were mainly attributable to lumbar disc reherniation (Table 3). Serious adverse events due to device deficiency were reported in 12 (4.4%) patients in the ACD group and included mesh migration (*n* = 5), mesh detachment (*n* = 3), anchor migration (n = 3), and anchor fracture (*n* = 1). Reherniation was identified at the time of reoperation in four of these patients. The frequency of all serious adverse events (Supplement Table 3) and all adverse events regardless of seriousness (Supplement Table 4) was comparable between groups.

**Table 3:** Device- or procedure-related serious adverse events through 3 years

Event	ACD (*n* = 272)			Control (*n* = 278)			*P* value
	Events	Patients	%	Events	Patients	%	
Any device or procedure-related SAE	39	29	10.7%	69	52	18.7%	0.008
Cardiac and vascular	0	0	0.0%	2	2	0.7%	0.499
Cardiac and vascular - other	0	0	0.0%	2	2	0.7%	
Device deficiency	11	11	4.0%	NA			NA
Anchor (whole device) migration	2	2	0.7%				
Mesh migration - extradiscal	4	4	1.5%				
Mesh migration - intradiscal	1	1	0.4%				
Mesh detachment - extradiscal	2	2	0.7%				
Mesh detachment - intradiscal	1	1	0.4%				
Anchor fracture	1	1	0.4%				
Disc herniation	20	17	6.3%	54	45	16.2%	< 0.001
Index level	18	15	5.5%	54	45	16.2%	
Residual herniation - index level	2	2	0.7%	0	0	0.0%	
Musculoskeletal - lumbar	0	0	0.0%	1	1	0.4%	1.000
Other	0	0	0.0%	1	1	0.4%	
Neurological - lumbar and lower extremity	1	1	0.4%	0	0	0.0%	0.495
Nerve or spinal root injury: index surgery	1	1	0.4%	0	0	0.0%	
Pain - lumbar and lower extremity	4	4	1.5%	4	2	0.7%	0.446
Lower extremity only	2	2	0.7%	3	2	0.7%	
Lumbar	1	1	0.4%	0	0	0.0%	
Lumbar and lower extremity	1	1	0.4%	1	1	0.4%	
Wound complication at index level	3	3	1.1%	8	6	2.2%	0.504
Dural injury/tear or CSF leak	1	1	0.4%	1	1	0.4%	
Infection	1	1	0.4%	3	2	0.7%	
Hematoma	0	0	0.0%	1	1	0.4%	
Delayed wound healing	1	1	0.4%	0	0	0.0%	
Dehiscence	0	0	0.0%	1	1	0.4%	
Deep	0	0	0.0%	2	2	0.7%	

*ACD*, annular closure device; *CSF*, cerebrospinal fluid leak; *NA*, not applicable; *SAE*, serious adverse event

## Discussion

A large unrepaired defect in the annulus fibrosus at completion of a lumbar discectomy procedure places patients at high risk for reherniation [13], which requires a reoperation in most cases to adequately resolve radicular symptoms [16]. In this randomized trial of high-risk patients, implantation with a bone-anchored ACD following limited lumbar discectomy reduced the risk of reherniation and reoperation and therefore achieved a better long-term pain and disability relief with associated higher levels of health-related quality of life compared with lumbar discectomy only. There were some specific risks to the ACD only, which included implantation difficulties, radiographic device deficiencies such as migration, mesh detachment, and VEPC. However, the overall safety profile was generally comparable between groups. These results suggest that lumbar discectomy with additional ACD implantation is an effective and safe procedure over 3-year follow-up in well-selected patients at high risk for reherniation owing to a large post-surgical annular defect.

Perhaps the most important finding of this trial relates to the ability of the ACD to lower the risk of recurrent herniation and reoperation among high-risk patients. This finding is notable given the previous failures of alternative annular defect closure methods such as sutures or fibrin glue [1, 2, 5, 7]. The fact that the mesh occlusion component is attached to an anchor within the vertebral body is the likely differentiating characteristic that offers a more durable annular occlusion. Several different mechanisms of device failure occurred, including mesh detachment, device migration, and anchor fracture. Most device failures were observed on follow-up imaging with no associated patient symptoms. However, device failure was associated with clinical sequelae in 4% of patients. All anchor migrations were observed within 3 months of surgery; two in patients with low regional bone density and one in a patient with multiple risk factors (heavy smoking, obesity, diabetes). It appears that device deficiencies, biomechanical forces, and/or patient characteristics may be causative factors involved in observed ACD failures.

Vertebral endplate changes detected on CT were more common among patients treated with ACD versus controls. While a detailed accounting of the association of VEPC with clinical outcomes is beyond the scope of this paper, the key findings warrant discussion. A noncontrolled study of 85 patients undergoing lumbar discectomy with or without additional ACD reported similar results where VEPC prevalence was higher in patients treated with ACD (52% vs. 10%), yet the reherniation rate on imaging irrespective of symptoms was lower with ACD (5% vs. 50%) [3]. Our group previously reported 2-year data from the current trial [10], which arrived at similar conclusions as with the current 3-year data. The presence of VEPC was not associated with patient-reported outcomes (leg pain, back pain, ODI) at 3 years after lumbar discectomy. Further, VEPC growth appears to be self-limiting with larger defects growing at the slowest rates. Based on the 3-year results of this trial, VEPCs occur more commonly with ACD, stabilize over time, and are not associated with adverse clinical sequelae.

Patient-reported outcomes statistically favored the ACD group at 3 years. However, the clinical implications of these findings are unclear since the mean differences between groups ranged from 0.3 to 0.6 MID units, depending on the outcome. A likely explanation for these modest differences is that this was a trial of a device intended to prevent, not treat, herniation recurrence. Since lumbar discectomy results in a durable surgical repair in 70–80% of cases, the benefit of an ACD would be realized only in the subsample of patients in which a reherniation was prevented but would not be anticipated to impact patient-reported outcomes otherwise. Based on the incidence of events and the risk reduction achieved with ACD, the number of patients needed to treat with ACD to prevent one symptomatic reherniation is 7; the number needed to treat to prevent one reoperation is 12.

Important strengths of this study were the multicenter randomized design, large sample size sufficient to detect even rare adverse events, and comprehensive independent review of imaging and adverse events during the trial. There are also several limitations of this study. First, these results are not applicable to all patients undergoing lumbar discectomy, but only the approximately 30% of cases at high risk of reherniation due to a large post-surgical annular defect [13]. The ACD is not intended to be used in patients with smaller defects since treatment with a permanent implant is difficult to justify in this population due to the relatively low risk of reherniation. Second, lack of patient and outcome-assessor blinding to treatment allocation may have biased patient-reported outcomes or the decision to reoperate. Third, while CT imaging with core laboratory reading is a strength of this trial, it may also be perceived as a limitation since the application of CT findings to routine clinical practice is unclear. Finally, longer follow-up is needed in this younger patient population to determine the durability of effect with ACD and to ensure there are no concerning late-onset safety- or device-related complications. While there was no association of VEPC with clinical complications over 3 years among patients who received ACD, this should be confirmed in long-term follow-up.

## Conclusion

The addition of a bone-anchored ACD to lumbar discectomy in patients with large post-surgical annular defects reduces the risk of reherniation and reoperation, with a better long-term pain and disability relief over 3-year follow-up compared with lumbar discectomy only. While the ACD was associated with distinct risks such as implantation difficulties, device migration, mesh detachment, and VEPC, the overall risk of complications was comparable between groups.

### Electronic supplementary material

ESM 1 (DOCX 64 kb)

## Appendix

**Table 4:** Study group appendix. List of investigators and participating centers

Site name	No. of patients enrolled	Principal investigator	Sub-investigator(s)
LKH Graz (Graz, Austria)	25	Sandro Eustacchio	Karin Pistracher Martin Trummer
ZNA Middleheim (Antwerp, Belgium)	48	Robert Hes*	Guido Dua
OLVG-location West (formerly: Sint Lucas Andreas Ziekenhuis) (Amsterdam, The Netherlands)	49	Gerrit J. Bouma	Michiel B. Lequin P. Richard Schuurman
St. Bonifatius Hospital GMbH (Lingen, Germany)	65	Peter Douglas Klassen	Bert Baume
Klinikum Augsburg (Augsburg, Germany)	20	Volkmar Heidecke	
Charite-Virchow-Klinikum (Berlin, Germany)	17	Peter Vajkoczy	Johannes Woitzik Marcus Czabanka
University Medical Center Schleswig-Holstein (Kiel, Germany)	25	Hubertus Mehdorn^‡^ Charlotte Flüh	Isabel Lübbing
OLV Ziekenhuis (Aalst, Belgium)	60	Frederic Martens	Geoffrey Lesage Djaya Kools
Universitätsmedizin Mannheim (Mannheim, Germany)	25	Jason Perrin	Mirko Arp Gregory Ehrlich
University Hospital Dϋsseldorf (Dϋsseldorf, Germany)	25	Richard Bostelmann	Jan Cornelius
Donauisar Klinikum Deggendorf (Deggendorf, Germany)	40	Stefan Rath	Mathias Haselbeck
Asklepios Westklinikum Hamburg (Hamburg, Germany)	13	Hans-Peter Köhler	Dirk Lympius Ekkehard von Saldern
Isala Ziekenhuis (Zwolle, The Netherlands)	17	Willem Art van den Brink	Dharmin Nanda
Medical University Innsbruck (Innsbruck, Austria)	25	Claudius Thomé	Pierre-Pascal Girod
A.Z Nikolaas Hospital (St. Nikolaas, Belgium)	5	Erik Van den Kelft	David Van der Planken
Haaglansden Medical Center: Antoniushove (Den Haag, The Netherlands)	19	Mark Arts	
Haaglanden Medical Center: Westeinde (Den Haag, The Netherlands)	1	Jasper Wolfs	
Kantonsspital Aarau (Aarau, Switzerland)	60	Javier Fandino	Jenny Kienzler
Knappschafts-Krankenhaus Bochum-Langendreer (Bochum, Germany)	8	Christopher Brenke	
Centre Hospitalier Régional Universitaire de Lille (CHRU), Hôpital Roger Salengro (Lille, France)	2	Richard Assaker	
Orthopädische Klinik und Poliklinik Universitätsmedizin Rostock (Rostock, Germany)	5	Susanne Fröhlich	Dorit Panser-Schulz

*PI moved to AZ Klina, Brasschaat, Belgium. EC approval at this site to follow patients enrolled at original site. PI at ZNA Middleheim now Guido Dua; ^‡^PI Mehdorn retired. Former Co-Investigator Jadik now PI
