# Supplementary material for: Three-year results from a randomized trial of lumbar discectomy with annulus fibrosus occlusion in patients at high risk for reherniation
Source: Acta Neurochir (Wien). 2019 May 15;161(7):1389–96. doi: 10.1007/s00701-019-03948-8 (PMC6581919; doi:10.1007/s00701-019-03948-8)
Supplement: Supplementary file 1 — (DOCX 64 kb) [file 701_2019_3948_MOESM1_ESM.docx]

**SUPPLEMENTARY TABLES**

**SUPPLEMENT TABLE 1**. Clinical Trial Inclusion and Exclusion Criteria.

**Inclusion Criteria**

1. Age 21 to 75 years and skeletally mature (male or female)

2. Posterior or posterolateral disk herniations at one level between L1 and S1 with confirmation of neural compression using MRI.[Note: Intraoperatively, only post-diskectomy anular defects between 4 and 6 mm tall and 6 and 10 mm wide shall qualify]

3. At least six weeks of failed, conservative treatment prior to surgery, including physical therapy, use of anti-inflammatory medications at maximum-specified dosage, and/or administration of epidural/facet injections

4. Minimum posterior disk height of 5mm at the index level

5. Radiculopathy (with or without back pain) with positive straight leg raise (0 –60 degrees) (L4/5, L5/S1) or femoral stretch test (L1/2, L2/3, L3/4 only)

6. Oswestry Disability Index score of at least 40/100 at baseline

7. Visual analog scale leg pain (one or both legs) score of at least 40/100 at baseline

8. Psychosocially, mentally, and physically able to fully comply with the clinical protocol and willing to adhere to follow-up schedule and requirements

**Exclusion Criteria**

1. Spondylolisthesis grade II or higher (25% slip or greater)

2. Requires spinal surgery other than a diskectomy (with or without laminotomy) to treat leg/back pain (scar tissue and osteophyte removal is allowed)

3. Back or non-radicular leg pain of unknown etiology

4. Prior surgery at the index lumbar vertebral level

5. Patients with a SCORE of 6 or greater and a subsequent spine DXA T-score less than -2.0 at the index level. For herniations at L5/S1, the average T-score of L1-L4 shall be used

6. Clinically compromised vertebral bodies in the lumbosacral region due to any traumatic, neoplastic, metabolic, or infectious pathology

7. Pathologicfractures of the vertebra or multiple fractures of the vertebra or hip

8. Scoliosis of greater than 10 degrees (both angular and rotational)

9. Any metabolic bone disease

10. Active infection, either systemic or local

11. Cauda equine syndrome or neurogenic bowel/bladder dysfunction

12. Severe arterial insufficiency of the legs or other peripheral vascular disease (Screening on physical examination for subjects with diminution or absence of dorsalis pedis or posterior tibialis pulses. If diminished or absent by palpation, then an arterial ultrasound is required with vascular plethysmography. Absolute arterial pressure below 50mm Hg at the calf or ankle level results in exclusion.)

13. Significant peripheral neuropathy, defined as Type I or II diabetes or similar systemic metabolic condition causing decreased sensation in a stocking-like or non-radicular and non-dermatomal distribution in the lower extremities

14. Insulin-dependent diabetes mellitus

15. Morbidly obese, defined as a body mass index > 40 or weighing more than 100 lbs over ideal body weight

16. Active hepatitis, AIDS, or HIV

17. Rheumatoid arthritis or other autoimmune disease

18. Known allergy to titanium, polyethylene, or polyester materials

19. Baseline MRI cannot be obtained

20. Pregnant or interested in becoming pregnant in the next three years

21. Active tuberculosis or history of tuberculosis in the past three years

22. History of active malignancy, defined as any invasive malignancy, except non-melanoma skin cancer, unless treated with curative intent with no signs or symptoms of malignancy for at least two years

23. Immunologically suppressed, defined as receiving steroids for more than one month over the past year

24. Current anticoagulation therapy other than aspirin, unless anticoagulation therapy may be suspended for surgery

25. Current chemical/alcohol dependency or significant psychosocial disturbance

26. Life expectancy of less than three years

27. Current involvement in active spinal litigation

28. Current involvement in another investigational study

29. Incarceration

30. Any contraindication for MRI or CT scan (e.g., claustrophobia, contrast allergy)

**SUPPLEMENT TABLE 2**. Study Assessments at Each Follow-up Interval.

| **Assessment** | **Pre-op** | **Surgery** | **6 weeks** | **3 months** | **6 months** | **1 year** | **2 year** | **3 year** |
| --- | --- | --- | --- | --- | --- | --- | --- | --- |
| Informed consent | ***** |  |  |  |  |  |  |  |
| Demographics/medical history | ***** |  |  |  |  |  |  |  |
| Pain medication | ***** | ***** | ***** | ***** | ***** | ***** | ***** | ***** |
| Clinical/neurologic exam | ***** | ***** | ***** | ***** | ***** | ***** | ***** | ***** |
| Neutral lateral/anteroposterior x-ray | ***** | ***** | ***** | ***** | ***** | ***** | ***** | ***** |
| Flexion-extension x-ray | ***** |  |  |  |  | ***** | ***** | ***** |
| Computed tomography | ***** |  |  |  |  | ***** | ***** | ***** |
| Magnetic resonance imaging | ***** |  |  |  |  | ***** | ***** | ***** |
| Randomization |  | ***** |  |  |  |  |  |  |
| Adverse events |  | † | † | † | † | † | † | † |

* Assessed at the specified study visit.

† Assessed continuously throughout the study.

**SUPPLEMENT TABLE 3.** Serious Adverse Events Through 3 Years.

| **Event** | **ACD**  **(n=272)** | | | **Control**  **(n=278)** | | | **p-value** |
| --- | --- | --- | --- | --- | --- | --- | --- |
|  | **Events** | **Patients** | **%** | **Events** | **Patients** | **%** |  |
| **Any SAE** | **156** | **93** | **34.2%** | **163** | **102** | **36.7%** | **0.593** |
| **Benign soft tissue masses/tumors** | **2** | **2** | **0.7%** | **2** | **2** | **0.7%** | **1.000** |
| **Cancer** | **3** | **3** | **1.1%** | **0** | **0** | **0.0%** | **0.120** |
| **Cardiac and Vascular** | **11** | **10** | **3.7%** | **5** | **5** | **1.8%** | **0.200** |
| Cardiac and Vascular - Other | 11 | 10 | 3.7% | 5 | 5 | 1.8% |  |
| **Death** | **1** | **1** | **0.4%** | **0** | **0** | **0.0%** | **0.495** |
| **Device Deficiency** | **12** | **12** | **4.4%** | **NA** | | | **NA** |
| Anchor (whole device) migration | 3 | 3 | 1.1% |  |  |  |  |
| Mesh Migration - Extradiscal | 4 | 4 | 1.5% |  |  |  |  |
| Mesh Migration - Intradiscal | 1 | 1 | 0.4% |  |  |  |  |
| Mesh Detachment - Extradiscal | 2 | 2 | 0.7% |  |  |  |  |
| Mesh Detachment - Intradiscal | 1 | 1 | 0.4% |  |  |  |  |
| Anchor Fracture | 1 | 1 | 0.4% |  |  |  |  |
| **Disc Herniation** | **31** | **26** | **9.6%** | **60** | **51** | **18.3%** | **0.003** |
| Herniation - Index Level | 21 | 18 | 6.6% | 57 | 48 | 17.3% |  |
| Residual herniation - Index Level | 2 | 2 | 0.7% | 0 | 0 | 0.0% |  |
| Disc Herniation - Adjacent Level | 2 | 2 | 0.7% | 3 | 3 | 1.1% |  |
| Disc Herniation - Non-Adjacent Level | 6 | 5 | 1.8% | 0 | 0 | 0.0% |  |
| **Endocrine** | **3** | **3** | **1.1%** | **0** | **0** | **0.0%** | **0.120** |
| **Eyes/Ears/Nose/Throat (EENT)** | **2** | **2** | **0.7%** | **8** | **8** | **2.9%** | **0.106** |
| **Gastrointestinal** | **11** | **8** | **2.9%** | **11** | **11** | **4.0%** | **0.642** |
| **Genitourinary** | **5** | **5** | **1.8%** | **5** | **5** | **1.8%** | **1.000** |
| **OB/GYN** | **6** | **6** | **2.2%** | **4** | **4** | **1.4%** | **0.541** |
| **Infectious Disease** | **2** | **2** | **0.7%** | **2** | **2** | **0.7%** | **1.000** |
| **Immunological** | **0** | **0** | **0.0%** | **1** | **1** | **0.4%** | **1.000** |
| **Metabolic/Hematologic/Electrolytes** | **0** | **0** | **0.0%** | **1** | **1** | **0.4%** | **1.000** |
| **Musculoskeletal - Lumbar** | **5** | **4** | **1.5%** | **3** | **3** | **1.1%** | **0.722** |
| Spinal Instability | 1 | 1 | 0.4% | 0 | 0 | 0.0% |  |
| Facet Syndrome | 4 | 3 | 1.1% | 1 | 1 | 0.4% |  |
| Other | 0 | 0 | 0.0% | 2 | 2 | 0.7% |  |
| **Musculoskeletal - Non-Lumbar** | **18** | **16** | **5.9%** | **15** | **13** | **4.7%** | **0.571** |
| **Neurological - Lumbar and Lower Extremity** | **3** | **3** | **1.1%** | **2** | **2** | **0.7%** | **0.683** |
| Nerve or Spinal Root Injury: Index Surgery | 1 | 1 | 0.4% | 0 | 0 | 0.0% |  |
| Nerve Root or Spinal cord Impingement | 1 | 1 | 0.4% | 0 | 0 | 0.0% |  |
| Musculoskeletal Spasms of the Back or Legs | 1 | 1 | 0.4% | 1 | 1 | 0.4% |  |
| Neurological Deterioration | 0 | 0 | 0.0% | 1 | 1 | 0.4% |  |
| **Neurological - Non-Lumbar/Lower Extremity** | **6** | **6** | **2.2%** | **8** | **6** | **2.2%** | **1.000** |
| **Pain - Lumbar and Lower Extremity** | **21** | **17** | **6.3%** | **15** | **11** | **4.0%** | **0.248** |
| Lower Extremity Only | 8 | 8 | 2.9% | 4 | 3 | 1.1% |  |
| Lumbar | 8 | 8 | 2.9% | 6 | 5 | 1.8% |  |
| Lumbar and Lower Extremity | 5 | 4 | 1.5% | 5 | 4 | 1.4% |  |
| **Psychological** | **2** | **2** | **0.7%** | **3** | **3** | **1.1%** | **1.000** |
| **Respiratory/Pulmonary** | **1** | **1** | **0.4%** | **5** | **5** | **1.8%** | **0.216** |
| **Trauma** | **8** | **8** | **2.9%** | **5** | **5** | **1.8%** | **0.414** |
| **Wound Complication at Index Level** | **3** | **3** | **1.1%** | **8** | **6** | **2.2%** | **0.504** |
| Dural Injury/Tear or CSF Leak | 1 | 1 | 0.4% | 1 | 1 | 0.4% |  |
| Infection | 1 | 1 | 0.4% | 3 | 2 | 0.7% |  |
| Hematoma | 0 | 0 | 0.0% | 1 | 1 | 0.4% |  |
| Delayed Wound Healing | 1 | 1 | 0.4% | 0 | 0 | 0.0% |  |
| Dehiscence | 0 | 0 | 0.0% | 1 | 1 | 0.4% |  |
| Deep | 0 | 0 | 0.0% | 2 | 2 | 0.7% |  |

ACD indicates annular closure device; NA, not applicable; SAE, serious adverse event.

**SUPPLEMENT TABLE 4.** Adverse Events Through 3 Years.

| **Event** | **ACD**  **(n=272)** | | | **Control**  **(n=278)** | | | **p-value** |
| --- | --- | --- | --- | --- | --- | --- | --- |
|  | **Events** | **Patients** | **%** | **Events** | **Patients** | **%** |  |
| **Any adverse event** | **565** | **222** | **81.6%** | **497** | **211** | **75.9%** | **0.118** |
| **Benign soft tissue masses/tumors** | **3** | **3** | **1.1%** | **2** | **2** | **0.7%** | **0.683** |
| **Cancer** | **5** | **5** | **1.8%** | **0** | **0** | **0.0%** | **0.029** |
| **Cardiac and Vascular** | **20** | **18** | **6.6%** | **16** | **15** | **5.4%** | **0.593** |
| Bleeding | 2 | 2 | 0.7% | 0 | 0 | 0.0% |  |
| Other | 18 | 16 | 5.9% | 16 | 15 | 5.4% |  |
| **Death** | **1** | **1** | **0.4%** | **0** | **0** | **0.0%** | **0.495** |
| **Dermatologic** | **4** | **4** | **1.5%** | **3** | **3** | **1.1%** | **0.722** |
| **Device Deficiency** | **34** | **33** | **12.1%** | **NA** | | | **NA** |
| Anchor (whole device) migration | 3 | 3 | 1.1% |  |  |  |  |
| Mesh Migration - Extradiscal | 8 | 8 | 2.9% |  |  |  |  |
| Mesh Migration - Intradiscal | 4 | 4 | 1.5% |  |  |  |  |
| Mesh Subsidence - no Detachment | 4 | 4 | 1.5% |  |  |  |  |
| Mesh Subsidence - with Detachment | 3 | 3 | 1.1% |  |  |  |  |
| Mesh Detachment - Extradiscal | 7 | 7 | 2.6% |  |  |  |  |
| Mesh Detachment - Intradiscal | 2 | 2 | 0.7% |  |  |  |  |
| Anchor Fracture | 2 | 2 | 0.7% |  |  |  |  |
| Other | 1 | 1 | 0.4% |  |  |  |  |
| **Disc Herniation** | **62** | **48** | **17.6%** | **99** | **82** | **29.5%** | **0.001** |
| Index Level | 37 | 32 | 11.8% | 88 | 75 | 27.0% |  |
| Residual herniation - Index Level | 2 | 2 | 0.7% | 0 | 0 | 0.0% |  |
| Adjacent Level | 15 | 14 | 5.1% | 8 | 8 | 2.9% |  |
| Non-Adjacent Level | 8 | 7 | 2.6% | 3 | 3 | 1.1% |  |
| **Endocrine** | **5** | **5** | **1.8%** | **2** | **2** | **0.7%** | **0.281** |
| **Eyes/Ears/Nose/Throat (EENT)** | **10** | **10** | **3.7%** | **17** | **17** | **6.1%** | **0.237** |
| **Gastrointestinal** | **25** | **19** | **7.0%** | **27** | **24** | **8.6%** | **0.527** |
| **Genitourinary** | **14** | **14** | **5.1%** | **10** | **9** | **3.2%** | **0.293** |
| **OB/GYN** | **7** | **7** | **2.6%** | **5** | **5** | **1.8%** | **0.573** |
| **Infectious Disease** | **4** | **3** | **1.1%** | **2** | **2** | **0.7%** | **0.683** |
| **Immunological** | **2** | **2** | **0.7%** | **6** | **6** | **2.2%** | **0.286** |
| **Metabolic/Hematologic/Electrolytes** | **4** | **4** | **1.5%** | **7** | **7** | **2.5%** | **0.545** |
| **Musculoskeletal - Lumbar** | **13** | **12** | **4.4%** | **11** | **9** | **3.2%** | **0.511** |
| Spinal Instability | 2 | 2 | 0.7% | 0 | 0 | 0.0% |  |
| Scoliosis | 0 | 0 | 0.0% | 3 | 3 | 1.1% |  |
| Radiographic Finding | 2 | 2 | 0.7% | 0 | 0 | 0.0% |  |
| Facet Syndrome | 9 | 8 | 2.9% | 5 | 4 | 1.4% |  |
| Other | 0 | 0 | 0.0% | 3 | 3 | 1.1% |  |
| **Musculoskeletal - Non-Lumbar** | **71** | **52** | **19.1%** | **64** | **53** | **19.1%** | **1.000** |
| **Neurological - Lumbar and Lower Extremity** | **41** | **36** | **13.2%** | **27** | **25** | **9.0%** | **0.135** |
| Nerve or Spinal Root Injury: Index Surgery | 3 | 3 | 1.1% | 3 | 3 | 1.1% |  |
| Nerve Root or Spinal cord Impingement | 3 | 3 | 1.1% | 1 | 1 | 0.4% |  |
| Musculoskeletal Spasms of the Back or Legs | 8 | 8 | 2.9% | 1 | 1 | 0.4% |  |
| Neurological Deterioration | 26 | 22 | 8.1% | 22 | 20 | 7.2% |  |
| Other | 1 | 1 | 0.4% | 0 | 0 | 0.0% |  |
| **Neurological - Non-Lumbar/Lower Extremity** | **20** | **18** | **6.6%** | **13** | **9** | **3.2%** | **0.077** |
| **Pain - Lumbar and Lower Extremity** | **115** | **84** | **30.9%** | **120** | **98** | **35.3%** | **0.279** |
| Lower Extremity Only | 35 | 33 | 12.1% | 44 | 40 | 14.4% |  |
| Lumbar | 58 | 51 | 18.8% | 65 | 60 | 21.6% |  |
| Lumbar and Lower Extremity | 21 | 19 | 7.0% | 11 | 10 | 3.6% |  |
| Non-specific | 1 | 1 | 0.4% | 0 | 0 | 0.0% |  |
| **Psychological** | **10** | **10** | **3.7%** | **8** | **8** | **2.9%** | **0.639** |
| **Respiratory/Pulmonary** | **4** | **4** | **1.5%** | **13** | **13** | **4.7%** | **0.046** |
| **Trauma** | **19** | **19** | **7.0%** | **19** | **17** | **6.1%** | **0.732** |
| **Wound Complication at Index Level** | **23** | **20** | **7.4%** | **22** | **19** | **6.8%** | **0.869** |
| Dural Injury/Tear or CSF Leak | 16 | 15 | 5.5% | 12 | 12 | 4.3% |  |
| Infection | 2 | 2 | 0.7% | 5 | 4 | 1.4% |  |
| Hematoma | 3 | 3 | 1.1% | 2 | 2 | 0.7% |  |
| Delayed Wound Healing | 1 | 1 | 0.4% | 0 | 0 | 0.0% |  |
| Dehiscence | 0 | 0 | 0.0% | 1 | 1 | 0.4% |  |
| Deep | 1 | 1 | 0.4% | 2 | 2 | 0.7% |  |
| **Site Reported Vertebral Endplate Change** | **49** | **48** | **17.6%** | **4** | **4** | **1.4%** | **<0.001** |

ACD indicates annular closure device; NA, not applicable.
